# Oral Acetazolamide after Boston Keratoprosthesis in Stevens Johnson Syndrome

**DOI:** 10.1186/1756-0500-5-205

**Published:** 2012-04-30

**Authors:** Radhika Kumar, Claes H Dohlman, James Chodosh

**Affiliations:** 1 Massachusetts Eye and Ear Infirmary, Harvard Medical School, Department of Ophthalmology, Boston, MA, USA

**Keywords:** Stevens Johnson syndrome, Toxic epidermal necrolysis, Acetazolamide, Glaucoma

## Abstract

**Background:**

Stevens-Johnson syndrome/toxic epidermal necrolysis (SJS/TEN) is a rare but severe and sometimes fatal condition associated with exposure to medications; sulfamethoxazole is among the most common causes. We sought to address the safety of acetazolamide, a chemically related compound, in patients with prior SJS/TEN and glaucoma. A retrospective case series is described of patients at the Massachusetts Eye and Ear Infirmary who underwent keratoprosthesis surgery for corneal blindness from SJS/TEN, and later required oral acetazolamide for elevated intraocular pressure.

**Findings:**

Over the last 10 years, 17 patients with SJS/TEN received a Boston keratoprosthesis. Of these, 11 developed elevated intraocular pressure that required administration of oral acetazolamide. One of 11 developed a mild allergic reaction, but no patient experienced a recurrence of SJS/TEN or any severe adverse reaction.

**Conclusion:**

Although an increase in the rate of recurrent SJS/TEN due to oral acetazolamide would not necessarily be apparent after treating only 11 patients, in our series, acetazolamide administration was well tolerated without serious sequela.

## Findings

### Background

Stevens Johnson syndrome and toxic epidermal necrolysis (SJS/TEN), two ends of a spectrum of severe adverse drug reactions affecting the skin and mucosa, typically result from hypersensitivity to a medication and manifest by acute sloughing of skin and mucous membranes. Secondary complications can be similar to burn patients with extensive loss of skin and mucosa, fever, hepatic, renal, and/or gastrointestinal involvement, sepsis, and death. Medications implicated in SJS/TEN include most commonly sulfonamides, anti-seizure medications, penicillins, and allopurinol, with onset of symptoms occurring within 1–8 weeks of drug administration [1-3]. SJS/TEN has an incidence of 1 to 6 cases per million person-years, and carries an overall mortality up to 15 % for SJS to 50 % for TEN. Ocular involvement during the acute phase occurs in up to 50-69 % of patients with SJS/TEN [4]. For survivors, severe dry eye, cicatricial ocular surface damage, and corneal blindness are feared outcomes. In one study, the prevalence of ocular complications of SJS was 20 % at 3 months following onset of SJS/TEN [5]. In our experience, however, patients with chronic ocular complications of SJS/TEN tend to worsen with time.

**Figure 1:**
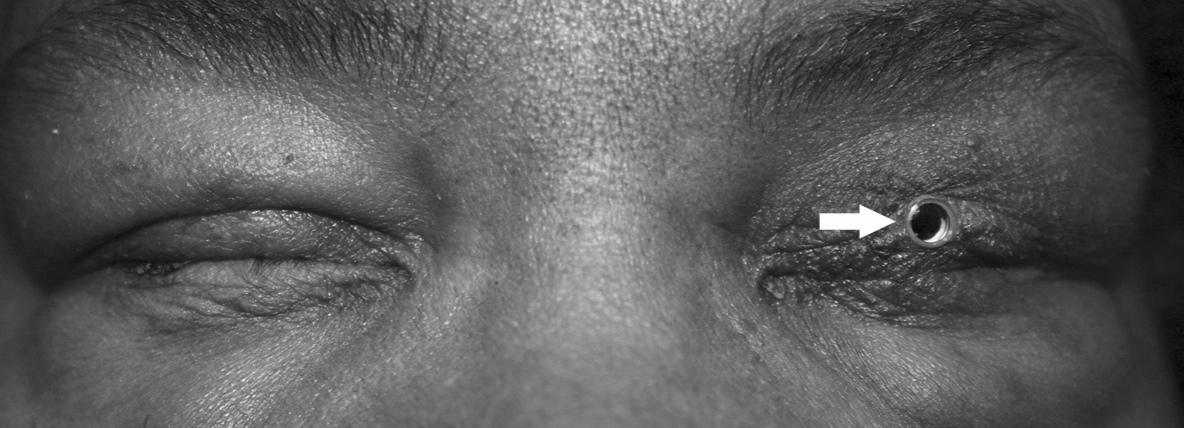
**Clinical appearance of the Boston type II keratoprosthesis in the left eye of a patient with history of toxic epidermal necrolysis.** Note the tight apposition between the eyelid skin and the keratoprosthesis stem (arrow).

In patients with loss of conjunctival fornices, keratinizing dry eye, and subsequent corneal blindness, the Boston keratoprosthesis type II, a collar button device of polymethylmethacrylate and titanium, is implanted to provide visual recovery [6]. However, postoperative glaucoma is common, even when a aqueous drainage device is implanted [7]. In Boston keratoprosthesis type II implantation, the eyelids are closed surgically around the prosthesis, as shown in Figure 1, after which, topical glaucoma drops have limited usefulness. Tube shunt failure after keratoprosthesis type II is common, and necessitates revision of the shunt or implantation of a second tube. Opening the fused eyelids for shunt revision or replacement, or for transcleral cyclophotocoagulation [7] may interfere with anatomic stability of the keratoprosthesis. Acetazolamide is a sulfonamide chemically distinct from sulfonamide antibiotics such as sulfamethoxazole. Use of oral acetazolamide or its sister drug methazolamide may be the only option in patients after Boston keratoprosthesis type II or after osteo-odonto keratoprosthesis, a different technique for restoration of end-stage cicatricial corneal blindness [8]. However, concerns over use of acetazolamide causing a second episode of SJS/TEN in keratoprosthesis patients have been raised [9], even when the drug initially associated with the patient’s SJS/TEN is not of the same chemical class.

Herein, we review the current literature pertaining to the use of sulfa-derivative medications in patients with a history of SJS/TEN, and present a series of patients at the Massachusetts Eye and Ear Infirmary in whom acetazolamide was used to treat intraocular pressure rises after implantation of a Boston keratoprosthesis.

## Methods

We performed a literature search using the search terms: Stevens-Johnson syndrome, toxic epidermal necrolysis, sulfa, Diamox, acetazolamide, allergy, and cross-reactivity. No articles were found that specifically addressed the safety of administration of sulfonamide nonantibiotics to patients with a history of SJS/TEN. With approval by the Human Studies Committee of the Massachusetts Eye and Ear Infirmary, we then performed a chart review of all patients with SJS/TEN who underwent Boston keratoprosthesis surgery from January 1, 2000 until December 31, 2010.

## Results

We identified 17 such patients; eleven (Table 1) were found to have received oral acetazolamide to reduce intraocular pressure at some point following their keratoprosthesis surgery. One patient had a type I keratoprosthesis (without eyelid surgery) but all others had a type II keratoprosthesis (eyelids surgically fused closed).

**Table 1:** Demographics of treated patients

**Pt. no.**	**Age at onset of SJS/TEN (years)**	**Inciting agent for SJS/TEN**	**Self-reported drug allergies**	**Time after surgery when acetazolamide started (months)**	**Presence of glaucoma drainage tube pre-acetazolamide**	**Time on acetazolamide (months)**	**Adverse reactions to acetazolamide**
1	43	ibuprofen	NSAID	1 day	Y	10 days	Warmth and pruritis
2*	48	sulindac	NSAID	24	N	1	N
3	40	allopurinol, erythromycin, colchicine	same	9	Y	9	N
4	50	Not known	terfenadine	0	N	31	N
5	3	PCN	PCN, sulfa, erythromycin	5	N	1.5	N
6	36	Not known	PCN, cefazolin, erythromycin	2	Y	24	N
7	54	bupropion, atenolol	same	13	Y	30	N
8	25	Not known	NKDA	7	Y	24	N
9	13	aspirin, ibuprofen	same	0.75	Y	1.5	N
10	Not known	Not known	PCN, codeine	Not known	Not known	9	N
11	27	PCN	PCN, sulfa	2	Y	64	N

*This patient received a Boston keratoprosthesis type I.

PCN: penicillin; NSAID: nonsteroidal anti-inflammatory drug.

Of the 11 patients who received oral acetazolamide, only one patient experienced an adverse reaction (warmth and pruritis); this occurred after 10 days of close monitoring. The affected patient never developed urticaria or dyspnea. Acetazolamide was stopped immediately upon development of symptoms, and no further adverse events occurred. It should be noted that the SJS/TEN in this patient was attributed to ibuprofen. The patient did not have a known allergy to acetazolamide, but had been told by her internist to avoid “sulfa” drugs due to their strong association with SJS/TEN. None of the patients in our series who were given acetazolamide reported sulfa as a cause of their SJS/TEN, but two reported the drug as an allergy. Interestingly, penicillin was thought to be the trigger for the SJS/TEN in both patients with sulfa allergy. Penicillin allergy seems to confer a heightened sensitivity to many other classes of medications [10]. Of the six patients who did not require oral acetazolamide, in three a sulfa drug caused their SJS/TEN, in two it occurred after treatment with penicillin, and in one patient the SJS/TEN was of unknown etiology (data not shown). All patients treated with oral acetazolamide demonstrated helpful reductions in intraocular pressure. In summary, acetazolamide was used in 11 SJS/TEN patients after keratoprosthesis, for a total of 16.3 person-years, with only one mild adverse reaction, and no reactivation of SJS/TEN.

## Discussion

SJS/TEN manifests clinically as mucocutaneous erythma and blistering, and leads to variable degrees of full thickness necrosis of skin and mucosae. The best available evidence suggests that SJS/TEN is drug metabolite specific, cytotoxic T cell-driven, and perforin/granzyme B triggered [3,11-18]. Specific HLA types associate with an increased risk of SJS/TEN to particular drugs [19-21]. In addition, fas and fas ligand have been implicated in the disorder, and may contribute to the characteristic expansion of keratinocyte apoptosis as the condition progresses [1,3,22]. Given the very real risk of death and the significant morbidity associated with SJS/TEN, and the disorder’s strong association with sulfonamides [2], controversy regarding the safety of acetazolamide in SJS/TEN is understandable. Furthermore, the rarity of SJS/TEN means that even a substantial increase in SJS/TEN incidence due to acetazolamide could be missed by individual physicians.

Although many different medications have been associated with SJS/TEN, the drug most commonly implicated is sulfamethoxazole [2], a sulfonylarylamine component of a commonly used combination antibiotic also containing trimethoprim. Re-administration of trimethoprim-sulfamethoxazole to a patient who previously developed SJS/TEN due to the same drug can cause recurrent SJS/TEN [23]. Numerous sources now support an absence of cross-reactivity between acetazolamide, a non-sulfonylarylamine, in patients with allergy to sulfamethoxazole [10,14,24,25].

Despite a common SO_2_NH_2_ group, there are two critical structural differences which distinguish sulfonamide antimicrobials such as sulfamethoxazole and sulfonamide nonantimicrobials such as acetazolamide; and these differences distinguish the drugs chemically and immunologically. Allergic reactions to sulfamethoxazole represent IgE-mediated immediate hypersensitivity reactions to the N1 heterocyclic ring, a structural component absent in acetazolamide [12,15]. Metabolite formation and subsequent specific cytotoxic T cell responses is stereospecific to the N4 amino nitrogen of sulfamethoxazole, also not found in acetazolamide [12,15].

Caution is indicated when prescribing any drug to a patient with prior SJS/TEN. However, in such patients acetazolamide should not be feared more than other drugs, even when the inciting agent for the original SJS/TEN was sulfamethoxazole. An interesting comparison may be made to celecoxib, another sulfonamide nonantibiotic [26]. Between January and June of 1999, 7 million prescriptions were written but only two possible cases of SJS/TEN were reported. If the risk of celecoxib causing SJS/TEN was similar to sulfamethoxazole, 7 million prescriptions would result in 30 to 70 cases of SJS/TEN [2,26]. This additional evidence supports a lack of cross reactivity induced by the two classes of sulfonamides.

In such a rare disorder as SJS/TEN, randomized clinical trials cannot be performed. Our retrospective case series includes too few patients to determine that acetazolamide is safe in patients with prior SJS/TEN. However, it is reassuring that we were able to safely administer acetazolamide to patients with a history of SJS/TEN, even the two patients reporting a “sulfa” allergy. We have not administered acetazolamide to any patient with sulfamethoxazole induced SJS/TEN. However, our case series is generally supportive of the use of acetazolamide in patients with a history of SJS/TEN, a population thought to have a higher incidence of drug-induced hypersensitivity.

## Conclusions

In conclusion, individuals with a history of SJS/TEN should be approached conservatively when it comes to administration of any medication. Safety measures such as skin testing, low test-dose administration of medication orally while under direct supervision, and HLA testing [21] may increase confidence in specific cases. In the setting of keratoprosthesis, oral acetazolamide may be the only medication short of glaucoma surgery which can lower intraocular pressure. We currently use oral acetazolamide in patients with SJS/TEN after implantation of the Boston keratoprosthesis type II as a temporizing measure for elevated intraocular pressure in the early postoperative period, and chronically in select patients with glaucoma shunt failure.

### Availability of supporting data

There is no supporting data beyond Table 1.

## Abbreviation

SJS/TEN: Stevens-Johnson syndrome/toxic epidermal necrolysis.

## Completing interests

The authors declare they have no competing interests or conflicts of interest associated with this manuscript.

## Authors’ contributions

James Chodosh accepts full responsibility for the work. RK and JC performed the literature review and patient chart review. RK, CHD, and JC drafted, read, and approved the manuscript.
